# Treatment outcome of tuberculosis at Dilla Referral Hospital, Gedeo Zone, southern Ethiopia: A retrospective study

**DOI:** 10.1371/journal.pone.0249369

**Published:** 2021-04-01

**Authors:** Endrias Markos Woldesemayat, Zewtir Azeze

**Affiliations:** 1 School of Public Health, College of Medicine and Health Sciences, Hawassa University, Hawassa, Ethiopia; 2 Digital Health Activity, John Snow Inc., Addis Ababa, Ethiopia; London School of Hygiene and Tropical Medicine, UNITED KINGDOM

## Abstract

**Background:**

Tuberculosis (TB) is one of the major public health problems in Ethiopia. Determining treatment outcome of TB cases could help to understand the effectiveness of TB control efforts. The objective of this study was to assess TB treatment outcome and associated factors and determine the risk factors of death among TB cases who were on Directly Observed Treatment Short course (DOTS).

**Methodology:**

We analyzed a retrospective data for TB cases who were on DOTS at Dilla Referral Hospital from July 2011- June 2016. The study population was TB cases with known HIV status and whose treatment outcome was evaluated at the Hospital. Data were entered, cleaned and analyzed using statistical package SPSS for windows, version 20.

**Result:**

Out of 899 registered TB cases, 731 included in this analysis. Of these cases, 424 (58.0%) were male and 334 (45.7%) were in the age group of below 25 years. Treatment success rate of TB was 675 (92.3%) and death rate was 18 (2.5%). Treatment outcome showed statistically significant variation by HIV status (P < 0.001). HIV status of the TB cases and pretreatment weight were associated with TB treatment outcome. HIV status of the TB cases was associated with death of the TB cases (Adjusted Odds Ratio (AOR) 5.0; CI 95%: 1.8–13.5).

**Conclusion:**

TB treatment success rate found in this study was high. Patient’s weight and HIV status were associated with treatment outcome. Moreover, HIV status predicted death of TB cases. Cautious treatment follow-up and defaulter tracing mechanisms for TB cases with these risk factors were suggested.

## Background

According to the World Health Organization (WHO) Global tuberculosis (TB) report, 10 million people were sick due to TB in 2019 while an estimated 1.4 million people died [1]. TB is a leading cause of morbidity and mortality particularly in the sub-Saharan Africa (SSA) region [2]. One of the TB control measures is effective implementation of the Directly Observed Treatment Short course (DOTS) strategy [3]. However, evaluating treatment outcome of TB cases who were on DOTS is also an important issue.

Ethiopia is one of the thirty TB high burden countries which accounts for almost 90% of those who fall sick with TB each year [1, 2]. The country has a total TB incidence rate of 140/10^5^ in 2019 [1]. The national population based TB prevalence survey conducted in 2010/11 revealed that the prevalence of smear-positive TB among adults and all age group was 108 and 63/10^5^ populations, respectively [4]. The prevalence of bacteriologically confirmed TB was 156/10^5^ populations and the prevalence for all forms of TB was 240/10^5^ populations in Ethiopia [4].

TB treatment outcome is important indicator in evaluating the effectiveness of performance of TB control programs [5]. Treatment of TB aims at curing the patient, interrupting transmission of mycobacterium TB and preventing bacilli from becoming drug resistant [6]. Unfortunately, these aims are not achieved in many countries which could affect achieving the post-2015 Global TB Strategy. In developing countries like Ethiopia, TB patients have multiple individual, disease specific and treatment related factors that adversely affect TB treatment outcomes [7]. Low treatment success rate threatens the effectiveness of TB control program which results in the development of multiple drug resistance TB (MDR-TB) [5].

In Hosaena, southern Ethiopia, out of 768 registered TB patients, 32.4% completed treatment, 10.9% cured 2.9% died and 0.5% fall in treatment failure category [8]. Of 1,172 participants involved in a study in Jinka, 13.1% cured, 60.9% completed their treatment and10.2% cases died during the course of treatment [9]. In Addis Ababa, Ethiopia certain study reported a 91.5% treatment success rate for TB [10]. Out of 339 patients in a treatment outcome analysis, 87.1% had successful treatment outcome in Debre Tabor, northern Ethiopia [11]. Another study carried out in Gonder Teaching Hospital showed that successful treatment was reported among 29.5% and death was reported among 10.1% of the TB cases [12]. A treatment success rate of 65.8% and death rate of 1.0% were reported in Nigeria [13]. In Ghana, an overall treatment success rate of 82.5% was reported, while death was reported for 13.5% of the cases [14]. In Zimbabwe, TB treatment success among adults TB patients was 73% [15].

A report from Hosana, in the southern Ethiopia showed that residence, TB category and HIV status were factors significantly associated with TB treatment outcomes [8]. The odds of successful treatment outcome was higher among patients ≥ 45 years of age and lower among females, rural residents, and patients with negative smear-result at the second month of treatment as compared to their counterparts in Debretabor, northern Ethiopia [11]. Being female, age group 15–24 years, smear-positive pulmonary TB and being urban resident were factors associated with higher treatment success rate in Gondar, northern Ethiopia [12]. Age and TB classification were factors significantly associated with TB treatment outcome in Addis Ababa, Ethiopia [10]. Factors like returned after treatment interruption and TB/HIV co-infection predicted poor treatment outcomes in another study in Ghana [14]. In Zimbabwe treatment success did not show difference when data were stratified by sex, age group and HIV status [15].

In Nigeria, 16.6% of the TB patients died with most deaths occurred soon after treatment onset. Risk factors for death of TB cases were being HIV-positive, residence outside the city, being treated for TB previously, absence of microbiological confirmation, having both pulmonary and extra-pulmonary TB and referral from a non-program linked clinics [16]. In Sidama, southern Ethiopia, more deaths occurred among smear-negative TB cases, cases older than 65 years and retreatment TB cases [17].

For years, DOTS has been under implementation in Gedeo Zone in the southern Ethiopia Region. A previous report in the specific setting [18] did not take in to consideration sero-status of the TB cases in the analysis. Expecting to fill the gap seen in the study, in the current study we included HIV status of TB cases which could affect the treatment outcome of TB. We also wanted to evaluate the changes in the proportion of treatment success after starting of HIV care in the hospital and determine the risk factors of death. The objectives of this study were to assess the treatment outcome and associated factors of TB treatment outcome and determine the risk factors of death among TB patients on DOTS at Dilla Referral Hospital, Gedeo Zone, South Ethiopia.

## Methods and materials

### Study area and period

The study was conducted in Gedeo Zone, which is located at a distance of 361 kilometers to the South of Addis Ababa, the country’s capital and 90 kilometers to the South of Hawassa city the capital of South Ethiopia Region. The Zone had a projected population of 1,139,429 million in 2017. Agriculture, mainly coffee cultivation is the predominant means of livelihood for residents in the Zone. Gedeo Zone has six districts and two town administrations with 35 health centers, three district Hospitals and one Referral Hospital. Data for this study were collected from Dilla Referral Hospital.

The hospital is located in Dilla Town, the capital of Gedeo Zone administration. It serves as referral center for patients referred from health centers, district hospitals and other private health facilities in Gedeo Zone and for patients coming from the neighboring Sidama and Oromia Regions. The hospital has a TB clinic where patients with TB are registered and treated based on DOTS strategy, according to the national TB cases treatment recommendation [19]. The DOTS strategy considers provision of the recommended medications for all registered TB cases, provider-initiated counseling and testing (PICT) service for all registered TB cases and antiretroviral therapy (ART) and linkage to ART for all eligible HIV-positive TB cases [19].

### Study design and population

In this study, we carried out a retrospective secondary data analysis. We reviewed TB treatment outcome data labeled on TB cases registry of Dilla Referral Hospital Hospital for the period from July 2011 to June 2016. The source population used in this study was all forms of TB patients who were diagnosed with active TB and put on DOTS at Dilla Referral Hospital TB clinic. The study population used in the study was all forms of TB cases with known HIV status and whose treatment outcome was evaluated at Dilla Referral Hospital. TB cases who were on DOTs for less than 4 weeks within the study period were excluded from the study. Sampling was not done to recruit the study participants. However, all TB patients who fulfilled the inclusion criteria were included in the study.

### Study variables

Treatment outcome of TB cases who were on DOTS at Dilla Referral Hospital was the outcome variable evaluated in the study. It included TB treatment outcomes like cured, treatment completed, died, treatment failure, defaulted treatment and moved to MDR-TB cases. Cured and treatment completed TB cases were considered as treatment success, while other treatment outcomes were considered as unsuccessful treatment outcomes. Socio-demographic characteristics like age, sex, residence and weight; clinical characteristics such as HIV status, TB type and category of TB treatment were independent variables measured in the study.

### Data collection

The data source for this study was Unit TB register at the TB clinic of Dilla Referral Hospital, where all patients diagnosed with active TB were put on anti-TB treatment regimens and monitored throughout the course of their treatment. Data were collected using a pre-tested data abstraction format. The format was developed considering variables to be studied that were described in the TB patients’ registration log book. We used two data collectors with a training background of diploma in nursing who were working in the hospitals TB clinic. A health officer who has been working in the hospital supervised the data collection. Data collectors and the supervisor received training on the format, inclusion and exclusion criteria of the study participants and recording of the right information from TB patients’ registration log book.

### Operational definitions

***Cured cases*** are pulmonary patients with bacteriological confirmed TB at the beginning of treatment who were smear and/or culture negative in the last month of treatment and on at least one previous occasion. ***Treatment completed cases*** refers to TB patients who completed treatment but are without evidence of treatment failure or with no records to show that sputum or culture results in the last month of treatment and on at least one previous occasion were negative, either because tests were not done or because results are unavailable. ***Treatment failure TB cases*** are TB cases whose sputum smear or culture were positive at month 5 or later during the course of treatment. ***Died cases*** are patients who died during the course of TB treatment irrespective of the cause. ***Lost to follow up*** was defined as patients who have been on treatment for at least four weeks and whose treatment was interrupted for eight or more consecutive weeks. ***Non evaluated cases*** are a TB cases for whom no treatment outcome was assigned. This includes “transferred out” to another treatment unit as well as cases for whom the treatment outcome was unknown. ***Transferred out cases*** refers to TB cases transferred out to another health facility or treatment unit. ***Moved to MDR-TB case*** refers to a patient who has been diagnosed as having DR-TB as per the national guideline and was placed on DR-TB treatment. ***Treatment success*** refers to the sum of cured TB cases and treatment completed TB cases. ***Unsuccessful treatment*** included died TB cases, treatment failure, moved to MDR-TB cases and lost to follow up TB cases. Operational definitions used in this study were used based on the National TB diagnosis and treatment guideline of Ethiopia [19].

### Data quality control

Data collectors and the supervisor were trained on issues related to the study. ZA delivered the training to enumerators and the supervisor. Data collection process was supervised. At the end of each data collection day, ZA and the supervisor checked completeness of the filled format. Data were cleaned.

### Data processing and analysis

Data entered, cleaned and analyzed using SPSS for windows version 20. A descriptive analysis was done for all variables included in the study. Descriptive statistics were determined using frequencies and percentages. Treatment outcome was recoded into two groups. Treatment outcomes; ‘Cured’ and ‘Treatment completed’ were grouped together as treatment success, favorable treatment outcomes. All other treatment outcomes were grouped together as unsuccessful treatment outcomes, unfavorable treatment outcomes.

Bivariate and multivariate logistic regression analyses were used to test the association between the independent variables and dependent variables (treatment outcome and death). Bivariate logistic regression analysis was done for variables like age, gender, residence, pretreatment weight, TB category and HIV status. Explanatory variables with P-value of less than 0.25 in the bivariate analysis were entered into multiple logistic regression models. For the bivariate and multivariate logistic regression analysis results Odds Ratio with the 95% confidence interval (CI) were described. P-value and 95% CI were reported to show the presence of statistical significance association between the dependent and independent variables.

### Ethical considerations

Ethical clearance was obtained from the Ethical Review Board (IRB) of Hawassa University College of Medicine and Health Sciences. After getting the ethical clearance, Hawassa University College of Medicine and Health Sciences wrote an official letter to Dilla Referral Hospital. Permission to carry out the study was obtained from the hospital after explanation of the purpose and benefit of the study. Patients’ medical records were accessed between November, 2017 and March, 2018. Data obtained were fully anonymous and only the authors had access to it.

## Results

### Demographic characteristics of patients and tuberculosis category

Between July 2011 and June 2016, a total of 899 TB cases were registered at Dilla Referral Hospital TB registry. Of these, 89 were transfer out cases, for 68 cases their treatment was not evaluated and 11 cases did not know their HIV status, and thus removed from analysis. Thus this study was based on 731 TB patients. Of these, 424 (58.0%) were male and 334 (45.7%) were in the age group of below 25 years. Majority, 601 (82.2%) of the TB cases were urban dwellers. About a quarter of the TB cases, 190 (26.0%) had a pretreatment body weight of at least 56 kg (Table 1).

**Table 1:** Demographic characteristics of tuberculosis patients at Dilla Referral Hospital, Gedeo Zone, South Ethiopia.

Characteristics	Frequency	Percent
**Age**		
≤14	98	13.4
15–24	236	32.3
25–34	210	28.7
35–44	101	13.8
45–54	44	6.0
≥ 55	42	5.7
**Sex**		
Male	424	58.0
Female	307	42.0
**Residence**		
Urban	601	82.2
Rural	130	17.8
**Pretreatment weight in kg**		
≤ 14	48	6.6
15–25	39	5.3
26–35	45	6.2
36–45	155	21.2
45–54	254	34.7
≥ 55	190	26.0

Kg = kilo gram

### Clinical characteristics

About three-fourth, 553 (75.6%) of the patients were pulmonary TB cases. Among the pulmonary TB cases, the majority was smear-negative pulmonary TB cases, which consisted of 307 (42.0%). Among the TB patients enrolled in the study, 124 (17.0%) were HIV positive. The proportion of TB cases in the retreatment category was 56 (7.7%) (Table 2).

**Table 2:** Clinical characteristics of tuberculosis patients at Dilla Referral Hospital, Gedeo Zone, South Ethiopia.

Variable	Frequency	Percent
**TB type**		
Pulmonary positive	246	33.7
Pulmonary negative	307	42.0
Extra pulmonary	178	24.4
**Treatment category**		
New	675	92.3
Retreatment	56	7.7
**HIV status**		
Positive	124	17.0
Negative	607	83.0
**ART treatment status**		
On ART	96	77.4
Not on ART	28	22.6

ART = antiretroviral therapy; TB = tuberculosis

### Tuberculosis treatment outcomes

Among TB patients involved in the study, treatment success rate was 675 (92.3%), with 165 (22.6%) cured and 510 (69.8%) treatment completed cases. Moreover, 13 (1.8%) TB cases had treatment failure, 18 (2.5%) were died cases, 20 (2.7%) were lost to follow up cases and 5 (0.7%) were moved to MDR-TB cases. TB treatment outcome showed statistically significant difference by HIV status (P < 0.001) (Table 3). The difference in pretreatment weight of TB cases for patients in the age group of ≤ 14 and 15 or more was not statistically significant (P > 0.05).

**Table 3:** Tuberculosis treatment outcomes by HIV status among TB patients at Dilla Referral Hospital, Gedeo Zone, South Ethiopia.

Category	HIV-positive	HIV-negative	Total	P-value
	n (%)	n (%)	n (%)	
Cured	24 (19.4)	141 (23.2)	165 (22.6)	< 0.001
Treatment completed	75 (60.5)	435 (71.7)	510 (69.8)	
Treatment failure	5 (4.0)	8 (1.3)	13 (1.8)	
Died	8 (6.5)	10 (1.6)	18 (2.5)	
Loss to follow up	10 (8.1)	10 (1.6)	20 (2.7)	
Moved to MDR-TB	2 (1.6)	3 (0.5)	5 (0.7)	
Successful outcome	99 (79.8)	576 (94.9)	675 (92.3)	< 0.001
Unsuccessful outcome	25 (20.2)	31 (5.1)	56 (7.7)	

MDR = multi-drug resistant; n = number; TB = tuberculosis

### Factors associated with tuberculosis treatment outcome and death

In a multivariate logistic regression analysis HIV status of the TB cases and TB treatment category showed association with TB treatment outcome. TB patients with positive HIV test results were over four times more likely to have the odds of unfavorable TB treatment outcome (Adjusted Odds Ratio (AOR) 4.3; CI 95%: 2.3, 8.1). TB patients with a pretreatment weight of below 15 kilograms had higher odds of unsuccessful TB treatment outcome (AOR 21.5; CI 95%: 3.4, 138.3). Table 4 shows results of factors associated with TB treatment outcome. HIV-positive TB cases had higher risk of death (AOR 5.0; CI 95%: 1.8–13.5) than HIV-negative TB cases. TB patients below 15 kilograms pretreatment body weight had higher risk of death compared to TB cases with a body weight of at least 55 kilograms (AOR 9.4; CI 95%: 1.5–60.8). However, the combined P-value for the assessment of association between pretreatment weight and death (which is a more informative one) showed absence of association (P = 0.29) (Table 5).

**Table 4:** Risk factors of unsuccessful treatment outcome of tuberculosis patients at Dilla Referral Hospital, Gedeo Zone, South Ethiopia.

	Treatment outcome		COR (95% CI)	P-value	AOR (95% CI)	P-value
	Unsuccessful	Successful				
**Age**						
≤14	8	90	1.8 (0.4, 8.7)	0.27	0.4 (0.0, 2.9)	0.45
15–24	11	225	1.0 (0.2, 4.8)		0.8 (0.2, 3.7)	
25–34	21	189	2.2 (0.5, 9.9)		1.3 (0.3, 5.9)	
35–44	11	90	2.4 (0.5, 11.5)		1.7 (0.3, 8.6)	
45–54	3	41	1.5 (0.2, 9.2)		1.2 (0.2, 8.2)	
≥ 55	2	40				
**Sex**						
Male	29	395	0.8 (0.4, 1.3)	0.33		
Female	27	280				
**Residence**						
Rural	10	120	0.9 (0.5, 2.0)	0.99		
Urban	46	555				
**TB type**						
Pulmonary positive	21	225	1.0 (0.5, 2.0)	0.61		
Pulmonary negative	20	287	0.8 (0.4, 1.5)			
Extra pulmonary	15	163				
**HIV status**						
Positive	25	99	4.7 (2.7, 8.3)	<0.01	4.3 (2.3, 8.1)	< 0.01
Negative	31	576	1		1	
**Pretreatment weight**						
< 14	6	42	4.4 (1.4, 14.3)	0.12	21.5 (3.4, 138.3)	0.02
15–24	4	35	3.5 (0.9,13.1)		11.9 (2.3, 62.9)	
25–34	6	39	4.7 (1.4,15.4)		5.8 (1.6, 20.2)	
35–44	13	142	2.8 (1.0,7.6)		3.0 (1.1, 8.4)	
45–54	21	233	2.8 (1.1,7.0)		3.1 (1.2,8.0)	
> 55		184	1		1	
**Treatment category**						
Re-treatment	9	47	2.6 (1.2, 5.5)	0.02	0.5 (0.2, 1.2)	0.14
New	47	628	1		1	

COR = Crude Odds Ratio; AOR = Adjusted Odds Ratio; CI = Confidence Interval

**Table 5:** Risk factors of death among tuberculosis patients at Dilla Referral Hospital, Gedeo Zone, South Ethiopia.

	Death		COR (95% CI)	P-value	AOR (95% CI)	P-value
	Yes	No				
**Age**						
≤14	3	95	1.3 (0.1, 12.8)	0.92		
15–24	5	231	0.9 (0.1, 7.8)			
25–34	4	206	0.8 (0.1, 7.3)			
35–44	4	97	1.7 (0.2, 15.6)			
45–54	1	43	0.9 (0.1, 15.7)			
≥ 55	1	41				
**Sex**						
Male	11	413	1.1 (0.4, 3.0)	0.79		
Female	7	300				
**Residence**						
Rural	4	126	1.3 (0.4, 4.1)	0.62		
Urban	14	587				
**TB type**						
Pulmonary positive	3	243	0.4 (0.1, 1.8)	0.32		
Pulmonary negative	10	297	1.2 (0.4, 3.5)			
Extra pulmonary	5	173				
**Treatment category**						
New	16	659	1			
Re-treatment	2	54	1.5 (0.3, 6.8)	0.58		
**HIV status**						
Negative	10	597	1		1	
Positive	8	116	4.1 (1.6, 10.7)	< 0.01	5.0 (1.8, 13.5)	< 0.01
**Pretreatment weight**						
≤ 14	3	45	6.3 (1.0, 38.6)	0.50	9.4 (1.5, 60.8)	0.29
15–24	1	38	2.5 (0.2,28.0)		4.7 (0.3, 35.2)	
25–34	1	44	2.1 (0.2,24.1)		2.0 (0.2, 22.5)	
35–44	5	150	3.1 (0.6,16.4)		3.2 (0.6, 16.8)	
45–54	6	248	2.3 (0.5,11.4)		2.3 (o.5, 11.6)	
≥ 55	2	188	1		1	

COR = Crude Odds Ratio; AOR = Adjusted Odds Ratio; CI = Confidence Interval

## Discussion

Evaluation of treatment outcome and associated factors of TB patients is important for assessing the effectiveness of DOTS program. In this study, the overall TB treatment success rate among TB cases involved in the study was high. The proportion of TB cases who died during the course of TB treatment was low. Pretreatment weight of patients and HIV status of the TB cases were factors associated with treatment outcome of TB. However, only HIV status of the TB patients showed association with death of TB cases.

The present study revealed that treatment success rate of TB was 92.3%. This rate was higher than a previous report in the same setting, 85.2% [18]. The variation could be related to improved accessibility of TB treatment in rural areas. In the previous report, 66% of study participants were rural dwellers [18], where as in the current study only 17.8% of the TB patients came from rural settings. Government interventions to improve the accessibility of TB care might have made many rural patients to receive TB care at health facilities found in their vicinity. In the previous report factors like residence and TB type were associated with treatment outcome of TB and only 11.5% of the study participants had extra pulmonary TB (EPTB) [18]. However, in the current study, 24% of the TB cases had EPTB and residence and TB type did not show statistically significant association with treatment outcome of TB. TB patients coming from towns have increased risk of having EPTB due to the high prevalence of HIV in urban areas. Improved accessibility of TB care in the study area might have minimized the risk of poor treatment outcome of TB.

Treatment success rate found in the current study was comparable to the report from Afar [20], Harar [21] and the national TB treatment success rate for new TB cases registered in 2017 [22]. In Harar, 30.4% were cured, 62.1% completed their treatment [21]. Among 400 studied TB cases at Enfaz Health Center, 379 (94.8%) had successful treatment outcome (302 treatment completed and 77 cured) [20]. The finding in the current study was above the report from Tetteh Quarshine Memorial Hospital of Ghana (75.4%) [23], Jinka (74%) [9], Addis Ababa (82.7%) [24], Adama (80.8) [25] and Hosana (43.3%) [8]. The higher treatment success rate observed in the current study could be explained by the improved access to and utilization of TB control services and improved awareness of patients about the disease. As majority of our study participants were urban dwellers, they had a better knowledge about TB and access to TB care providing facilities. The studies from Ghana, Jinka and Addis Ababa [5, 23, 24] did not specify where their study participants came from, while the studies from Adama and Hosana had low proportion of their study participants came from Urban settings [8, 25] compared to the 82.2% urban dwellers involved in the current study. The difference in geographical location and health institution setting may also contribute to the observed differences.

Our data showed the absence of statistically significant difference in pretreatment weight among children and adults. Therefore, we did not report age stratified analysis for pretreatment weight. However, patients with higher pretreatment weight had increased chance of having treatment success. The study from Harar Town confirmed the association between pretreatment weight and TB treatment outcome [21]. Low pretreatment weight of TB patients may indicate advanced stage of the disease, which may cause poor treatment outcome. For improving treatment outcome, we suggest TB patients with low pretreatment weight demand more care during the course of TB treatment.

M. Endris and his colleagues reported absence of significant association between HIV status and successful TB treatment outcome [20]. In contrary to this, our analysis showed that, HIV negative TB cases had higher treatment success than HIV positive TB cases. This finding is in line with the study report from Nigist Elleni hospital, which showed that HIV positive TB patients had high risk of poor treatment outcomes compared to HIV negative TB patients [8]. Studies from other settings also confirmed such an association [9, 21, 25, 26]. Our data showed that, compared to HIV negative TB cases, high proportion of TB/HIV co-infected cases had poor treatment outcome; such as lost follow-up (8.1%), treatment failure (4.0%) and death (6.5%). For improving TB treatment outcome, it is important doing strong follow-up and defaulter tracing mechanisms for TB patients with HIV positive status.

In contrary to the finding from Hosana [8], treatment category of TB cases did not predict treatment success at Dilla. Compared to new TB cases, retreatment TB cases face poor TB treatment outcome [8]. In a crude analysis of our data, TB treatment category showed statistically significant association with poor TB treatment outcome. However, the association did not maintain in the adjusted analysis.

There was no significant association between sex or age of TB cases with TB treatment outcome [20]. However, other studies reported the association between age and successful TB treatment outcome [18, 21, 25]. Unlike the findings in these studies, [18, 21, 25], age of TB patients was not associated with treatment outcome among our study participants. Moreover, gender of patients was not associated with TB treatment outcome. Our finding is in line with a study report from Nekemete Referral Hospital in West Ethiopia [26].

Compared to the findings in many other settings [9, 17, 20, 21, 24, 26], the mortality rate observed in the current study was low. Unlike what Feng JY and et al reported [27], in the current study, we found no significant difference in mortality by gender and residence. The absence of such an association in the current study could be explained by improved access and utilization of TB control services and an improved awareness of the population about the disease. As expected, our data showed the existence of increased risk of death among HIV positive TB patients. Similar results were reported in other settings [16, 28]. During follow up of TB cases on treatment it is important to give attention for TB cases with comorbidity (HIV). This could minimize the rate of death among them.

This study was done using secondary data from a unit TB register, so it was not possible to collect additional data on important characteristics such educational status of the study participants which could affect treatment outcome of TB. In the study, omitting transfer out TB cases, the non-evaluated TB cases and TB cases with unknown HIV status might have affected the results. Absence of study participants’ height to estimate body mass index (BMI) is another limitation of this study. It is difficult to correlate treatment outcome with body weight alone as it cannot inform the body condition of an individual unless it is correlated with age or height. So we suggest readers to interpret the findings of our study in light of the stated limitations.

## Conclusion

This study evaluated treatment outcome of TB patients on DOTs and identified factors associated with TB treatment out come and death of TB cases. The level of treatment success observed in the study was encouraging. HIV positive status and pretreatment patient weight were factors independently associated with unsuccessful TB treatment outcome. HIV-positive status of the TB patients predicted death. For maintaining the current treatment success observed in this study and to minimize the risk of death, it is important giving attention to HIV positive TB cases and patients with low pretreatment weight. Cautious follow-up and defaulter tracing mechanisms of these cases are suggested.

## Supporting information

S1 Dataset A data set for treatment outcome of tuberculosis at Dilla Referral Hospital, Gedeo Zone, South Ethiopia. (CSV)
